# Are we getting late to feed the patients after opening percutaneous endoscopic gastrostomy in the intensive care unit?

**DOI:** 10.1186/2197-425X-3-S1-A576

**Published:** 2015-10-01

**Authors:** BB Yelken, E Ozen, CA Uzan, S Ekemen

**Affiliations:** Eskisehir Osmangazi University Medical Faculty, Anesthesiology and ICU, Eskisehir, Turkey

## Introduction

Percutaneous endoscopic gastrostomy (PEG) has becoming the most common way of feeding patients who cannot take adequate oral nutrition in the intensive care unit (ICU). According to ESPEN guidelines after complication-free placement of a PEG system,delivery of nutrients via the tube can commence within 1 hour. But traditionally many clinicians are waiting 24 or more hours to start feeding their patients.

## Objectives

The aim of our study is to determine when we start to feed the patients and when we reach maximal enteral feeding dose after PEG placement in our ICU. So we can answer the question if we are getting late or not.

## Methods

We collected information about 20 patients who underwent PEG without any complication in our ICU. All the PEG's were performed with pull-through technique in the general surgery operation room under deep sedation

## Results

Twenty patients data was collected :9 was female and 11 was male. Mean age of the patients was 49.6.The patients have different range of diagnoses like carbonmonoxide intoxication, polytrauma, tetanus. The earliest time to start enteral feeding via PEG was 4 hours and the latest was 74 hours. The earliest time to reach maximum enteral feeding dose was 31 hours and the latest was 204 hours. The mean time to start feeding was 26.1 hours and the mean time to reach maximal enteral feeding dose was 125.85 hours.

## Conclusions

We found that we are getting late to feed the patients in our ICU after PEG placement. We should encourage our ICU staff to start feeding early.

## Grant Acknowledgment

The content is solely the responsibility of the authors and was not supported by any funding, organisation etc.

## References

[1] Best C (2009). Percutaneous endoscopic gastrostomy feeding in the adult patient. Br J Nurs.

[2] Martín A, Espinós JC, Forné M, Rius J, Corbera G, Quintana S, Viver JM (1994). [Percutaneous endoscopic gastrostomy: study on 35 patients]. Med Clin (Barc).

[3] Islek A, Sayar E, Yilmaz A, Artan R (2013). Percutaneous endoscopic gastrostomy in children: Is early feeding safe?. J Pediatr Gastroenterol Nutr.

